# Usefulness of Hydrogel-Coated Coils in Embolization of Pulmonary Arteriovenous Malformations

**DOI:** 10.1007/s00270-018-1876-5

**Published:** 2018-01-17

**Authors:** Masashi Shimohira, Tatsuya Kawai, Takuya Hashizume, Masahiro Muto, Masanori Kitase, Yuta Shibamoto

**Affiliations:** 1 Department of Radiology, Nagoya City University Graduate School of Medical Sciences, Nagoya, 467-8601 Japan; 2 Department of Radiology, Nagoya City East Medical Center, Nagoya, 464-0071 Japan; 3 Department of Radiology, Kariya Toyota General Hospital, Kariya, 448-8505 Japan

**Keywords:** Pulmonary arteriovenous malformation, Embolization, Hydrogel-coated coil

## Abstract

**Purpose:**

To evaluate the usefulness of hydrogel-coated coils for preventing recanalization after coil embolization of pulmonary arteriovenous malformations (PAVMs).

**Materials and Methods:**

Thirty-seven consecutive patients with 57 untreated PAVMs underwent coil embolization with hydrogel-coated coils between January 2013 and Jun 2017. The mean age was 49 years (range 9–83 years), and there were seven male patients and 30 female patients. The median size of the feeding artery was 3.7 mm (range 1.5–6.1 mm), and the median size of the venous sac was 9.3 mm (range 2.6–36.6 mm). For all PAVM, embolization was attempted using 0.018-in. hydrogel-coated coils with or without other coils (0.0135–0.018-in. bare platinum coils and fibered platinum coils). Technical success rate, recanalization rate, and complications were evaluated. Technical success was defined as completion of embolization using hydrogel-coated coils. Recanalization was evaluated with time-resolved magnetic resonance angiography and/or pulmonary angiography.

**Results:**

In 56 of 57 PAVMs, embolization was successfully performed with hydrogel-coated coils. Therefore, the technical success rate was 98% (56/57). The number of PAVMs at risk was 56, 42, 18, and 12 at 0, 12, 24, and 36 months, respectively. There was no recanalization with a mean follow-up period of 19 months (range 2–47 months) in 56 PAVMs embolized with hydrogel-coated coils. There were no major complications. As a minor complication, local pain was observed in 8 of 43 sessions (19%) after embolization.

**Conclusions:**

Hydrogel-coated coils may be useful for preventing recanalization after the embolization of PAVMs.

## Introduction

Pulmonary arteriovenous malformations (PAVMs) are abnormal fistulas that appear between the pulmonary arteries and veins that bypass the normal capillaries [1, 2]. In this condition, the lung loses its ability to filter emboli and bacteria, which can then pass directly into the systemic circulation, causing stroke or cerebral abscess [3]. Coil embolization is widely performed for PAVMs; however, persistence of PAVM can be a problem after embolization. This has been attributed to recanalization, in which PAVMs are perfused due to flow through a previously placed coil nest; pulmonary-to-pulmonary reperfusion, in which the embolized feeder remains occluded but there are small feeders from adjacent normal pulmonary arteries; incomplete primary treatment, in which there are previously untreated feeders of a complex PAVM; and systemic-to-pulmonary reperfusion, in which PAVMs persist via a systemic arterial feeder but are not seen on pulmonary arteriography [2]. Among these causes of persistence, recanalization is the most frequent cause [2], and it can lead to the risk of stroke or cerebral abscess. To prevent recanalization, the feeding artery of a PAVM should be embolized tightly [4].

Meanwhile, hydrogel-coated coils consist of a layer of hydrogel polymer surrounding a platinum metal core and can fully expand within 20 min upon contact with blood [5]. There is a nearly fivefold higher filling volume for the 0.018-in. coil than for platinum coils of the same size, and they have mostly been used in neurointerventional procedures and are useful for preventing recanalization of cerebral aneurysms [6–8].

The purpose of this study was to evaluate the usefulness of hydrogel-coated coils in preventing recanalization after coil embolization of PAVMs.

## Materials and Methods

This retrospective study was approved by an institutional review board. Each patient provided written informed consent to undergo the procedure. Between January 2013 and Jun 2017, 37 consecutive patients with 57 untreated PAVMs underwent coil embolization with hydrogel-coated coils. According to a recent article [9], coil embolization was attempted for all PAVMs diagnosed as accessible in CT regardless the diameter of the feeding artery. All PAVMs could be followed after embolization and were included in this study. The mean age was 49 years (range 9–83 years), and there were seven male patients and 30 female patients. Fifty PAVMs had a single feeding artery (simple type), and 7 PAVMs had multiple feeding arteries (complex type). The median size of the feeding artery was 3.7 mm (range 1.5–6.8 mm), and the median size of the venous sac was 9.3 mm (range 2.6–36.6 mm). Details of the patients and PAVMs are summarized in Table 1. For all PAVM, embolization was attempted using 0.018-in. hydrogel-coated coils with or without other coils: 0.0135–0.018-in. bare platinum coils and fibered platinum coils. In four patients, 3 PAVMs were embolized in one session, and in five patients, 2 PAVMs were embolized in one session. Thus, the total session number was 43. All procedures were performed by a single operator (M.S.), who had 14 years of experience in diagnostic and interventional radiology, with assistants (T.H., M.M.).

**Table 1:** Details of patients and PAVMs

Patients (*n* = 37)	Male/female	7/30
	Age#	49 (9–83)
PAVMs (*n* = 57)	Type	
	Simple/complex	50/7
	Location	
	RUL/RML/RLL/LUL/LLL	4/14/19/9/11
	Size^b^	
	Feeding artery (mm)^c^	3.7 (1.5–6.8)
	Venous sac (mm)	9.3 (2.6–36.6)
	Follow-up period (months)^a^	19 (2–47)

^a^Values are reported as mean (range)

^b^Values are reported as median (range)

^c^The size of the largest feeding artery was used for the complex type

*RUL* right upper lobe, *RML* right middle lobe, *RLL* right lower lobe, *LUL* left upper lobe, *LLL* left lower lobe

Medical records and imaging were reviewed, and technical success rate, recanalization rate, details of used coils (type, number, length, and volume) and complications were evaluated. Technical success was defined as completion of embolization for PAVM using hydrogel-coated coils. Recanalization was evaluated with time-resolved magnetic resonance angiography (TR-MRA) and/or pulmonary angiography (PAG). Coil volume was calculated assuming a cylindrical coil shape using the formula: coil volume = *π* × (coil radius)^2^ × (coil length) for each coil. In this calculation, the volume of the hydrogel-coated coils was calculated assuming full hydrogel polymer expansion. Complications that required extended hospitalization or an advanced level of care or resulted in permanent adverse sequelae or death were classified as major complications, and the remaining complications were considered minor [10].

### Coil Embolization Technique

All procedures were approached via the femoral vein with an 8-Fr sheath (SuperSheath; Medikit, Tokyo, Japan). To prevent the formation of a thrombus during the procedure, 3000 units (1000 units/mL) of heparin were administered intravenously, with an additional 1000 units added every hour. The triaxial system was introduced for the procedure. An 8-Fr balloon catheter (Optimo; Tokai Medical, Kasugai, Japan) was placed at the segmental pulmonary artery, followed by a 4-Fr catheter (Multi-purpose; Terumo, Tokyo, Japan, or Cerulean G; Medikit) and a 2.2-Fr microcatheter (Progreat β3; Terumo). Saline flushing was performed for each lumen during the procedure to prevent thrombus formation. An 8-Fr balloon catheter was used to occlude the blood flow of PAVM to prevent paradoxical embolization and migration of coils to the venous side and also as a backup in case of perforation. Deep advancement of the 4-Fr catheter was attempted for good support of the microcatheter. Coil embolization was attempted using hydrogel-coated coils (AZUR; Terumo), bare coils (IDC; Boston Scientific Corporation, Natick, MA or Target; Stryker, Fremont, CA) and/or fibered coils (Interlock; Boston Scientific Corporation, or Trufill; Codman & Shurtleff, Raynham, MA). All used coils were chosen by the operator (M.S.).

In all procedures, embolization was performed in the following manner. At first, the microcatheter was advanced to the entrance of the venous sac, and several coils were placed until the operator could feel slight resistance to make a scaffold in venous sac. Then, the microcatheter was pulled back to the distal feeding artery and embolization was continued. To make tight packing of the distal feeding artery, we attempted to place the hydrogel-coated coils at the distal feeding artery instead of the venous sac. It was also because hydrogel-coated coils were relatively stiff and had a possibility of venous sac injury.

### Follow-Up Examinations for Recanalization

Follow-up examinations including TR-MRA were basically planned at 2, 6, and 12 months, and every 12 months thereafter, but it was adjusted according to subsequent embolization sessions in patients with multiple PAVM and individual patients’ situations. The median number of follow-up examinations was 3 (range 1–7), and the median periods of the first, second, third, fourth, fifth, sixth, and seventh follow-up examinations were 2, 6, 12, 18, 29, 32, and 41 months after coil embolization. On TR-MRA, recanalization was defined as simultaneous enhancement of the feeding artery and draining vein or enhancement of the venous sac in the pulmonary arterial phase (before visualization of the normal pulmonary vein) [11–14]. In patients with multiple PAVMs, not all of the PAVMs could be embolized in a single session; thus, those that were previously embolized were evaluated by using PAG during subsequent embolization sessions, instead of TR-MRA. PAG was only performed in patients who required additional embolization, which was planned 1–4 months after the first session. Recanalization was defined as simultaneous enhancement of the feeding artery and draining vein or the venous sac [13]. Follow-up images were interpreted for recanalization by two radiologists (M.S. and T.K., with 14 and 12 years of experience, respectively, in diagnostic and interventional radiology). Any discrepancies were resolved through consensus.

### Image Acquisition and TR-MRA Reconstruction

All TR-MRAs were performed on a 1.5-T MR system (Achieva; Philips Healthcare, Best, The Netherlands) or a 3.0-T system (Skyra; SIEMENS, Erlangen, Germany, Ingenia; Philips Healthcare). TR-MRA was acquired using a 3D-T1-weighted fast-field echo sequence; the parameters were based on a previously reported method [13]. The achieved temporal resolution was 1.7 s for Achieva and Ingenia and 1.0 s for Skyra. Consecutive 3D volume images (25 images for Achieva and Ingenia and 30 images for Skyra) were acquired immediately after injection of contrast media (0.1 mmol/kg Gd-chelate, Magnevist; Bayer HealthCare, Whippany, NJ, USA) at a flow rate of 2 mL/s, followed by a saline flush of 30 mL during breath-holding. All source images from each frame were reconstructed with a maximum intensity projection algorithm. Both source images and their maximum intensity projection images were evaluated to diagnose recanalization.

## Results

**Fig. 1:**
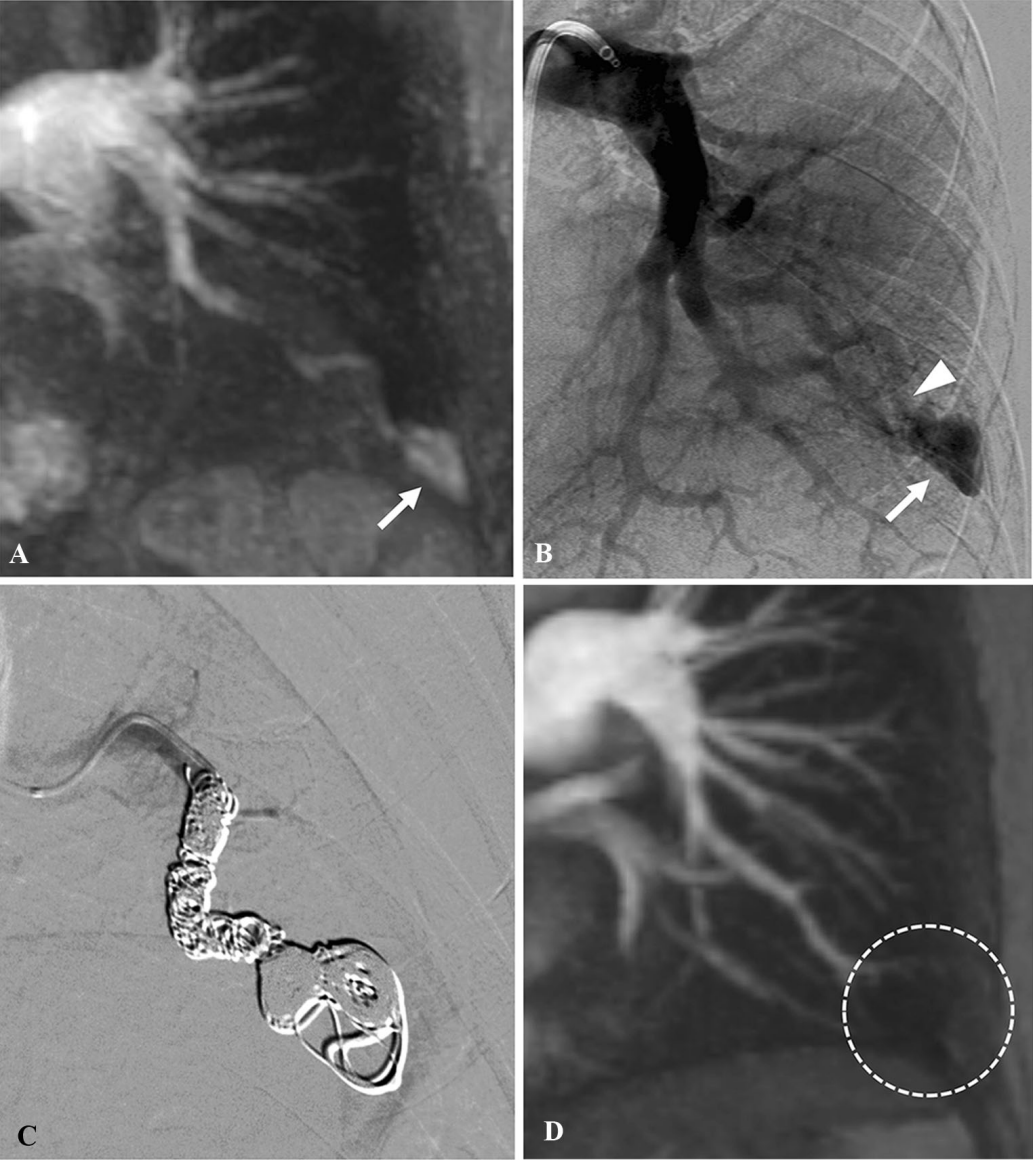
A 57-year-old woman presented with untreated PAVM of the left lower lobe. **A** Time-resolved magnetic resonance angiography (TR-MRA) shows a PAVM in the left lower lobe (arrow). **B** Angiography of the left pulmonary artery shows a PAVM (arrow); then the feeding artery from the anteromedial basal segmental artery (arrow head) was selected and embolization was performed with bare coils, fibered coils, and hydrogel-coated coils. **C** Angiography after the embolization shows complete occlusion of the PAVM. **D** TR-MRA 28 months after coil embolization shows no recanalization

**Fig. 2:**
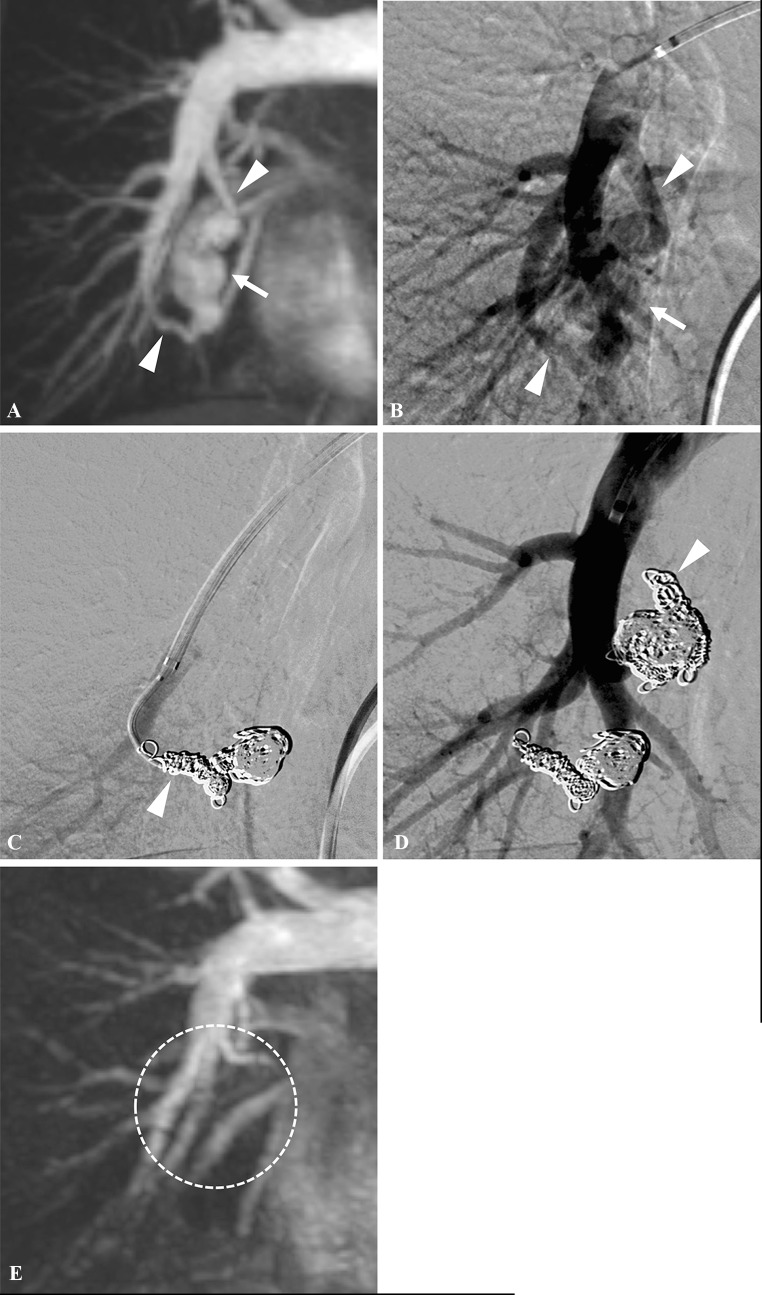
A 40-year-old woman presented with untreated complex PAVM of the right lower lobe. **A** Time-resolved magnetic resonance angiography (TR-MRA) shows a PAVM in the right lower lobe (arrow) with two feeding arteries (arrow heads). **B** Angiography shows the PAVM (arrow) with two feeding arteries (arrow heads). **C** First, a feeding artery from the anterior basal segmental artery (arrow head) was selected, and then, embolization was performed with bare coils, fibered coils, and hydrogel-coated coils. **D** Second, another feeding artery from the medial basal segmental artery (arrow head) was selected, and embolization was performed in the same manner. **E** TR-MRA 21 months after coil embolization shows no recanalization

In 56 of 57 PAVMs, embolization was successfully performed with hydrogel-coated coils. Therefore, the technical success rate was 98% (56/57). However, in one patient with two PAVMs, the placement of a hydrogel-coated coil failed for one of the PAVMs, because, although the diameter of the feeding artery was 2 mm, the minimum size of hydrogel-coated coils in our country was 3 mm at that time. The coil could not be placed successfully and retrieved. So, embolization was performed without hydrogel-coated coils, and bare coils and fibered coils were used instead; thereafter, no persistence was observed with 43 months follow-up. The number of PAVMs at risk was 56, 42, 18, 12, and 0, at 0, 12, 24, 36, and 48 months, respectively. In 56 PAVMs embolized with hydrogel-coated coils, there was no recanalization with a mean follow-up period of 19 months (range 2–47 months) (Figs. 1, 2). Furthermore, there was no other type of persistence such as pulmonary-to-pulmonary reperfusion, incomplete primary treatment, and systemic-to-pulmonary reperfusion during this period. The used coils are as follows: hydrogel-coated coils, bare coils, and fibered coils in 47; hydrogel-coated coils and bare coils in 5; hydrogel-coated coils and fibered coils in 3; and hydrogel-coated coils only in 1. The coil details are shown in Table 2. The median number of hydrogel-coated coils was 4, the median total length of the hydrogel-coated coils was 60 cm, and the median volume of hydrogel-coated coils was 342 mm^3^. The median percentage of hydrogel-coated coils used was 32% (range 10–100%) in number, 35% (8.4–100%) in length, and 75% (32–100%) in volume.

**Table 2:** Details of coils used for PAVMs embolized with hydrogel-coated coils

	Total	Bare	Fibered	Hydrogel
Number	13 (4–49)	4 (0–25)	5 (0–30)	4 (1–17)
%		25 (0–76)	40 (0–77)	32 (10–100)
Length (cm)	168 (20–947)	85 (0–835)	13.5 (0–181)	60 (4–239)
%		47 (0–88)	8.2 (0–51.6)	35 (8.4–100)
Volume (mm^3^)^a^	462 (41–1997)	86 (0–1352)	12 (0–187)	342 (23–1400)
%		19 (0–68)	30 (0–25)	75 (32–100)

All figures are median and range

^a^For calculation of hydrogel-coated coil volumes, full expansion of the coils was assumed

There were no major complications. Local pain, as a minor complication, was observed in 8 of 43 sessions (19%) after embolization and was controllable with an analgesic. Overall, the mean follow-up period was 19 months (range 2–47 months). Of the 56 PAVMs, 41 were monitored using TR-MRA only, and 15 were monitored with both TR-MRA and PAG. One patient each was lost after the follow-up TR-MRA at 2, 2, 2, and 17 months, with no recanalization or other persistence. The remaining 33 patients were continuously followed.

## Discussion

In the present study, there was no recanalization in all PAVMs embolized with hydrogel-coated coils. We think the expanded hydrogel polymer contributes to increased coil volume and tightly embolizes the PAVM. In the literature, higher packing density was reported with hydrogel-coated coils than with bare coils in embolization of elastase-induced saccular aneurysms in rabbits. Furthermore, histological analysis of aneurysms embolized with hydrogel-coated coils indicated that most of the aneurysm cavity was filled with expanded hydrogel [15]. Thus, in our study also, we believe there was a considerable amount of expanded hydrogel polymer in the feeding artery of the PAVMs, which could achieve tight mechanical occlusion without the aid of thrombus formation; as a result, recanalization was not likely. Furthermore, it was reported that hydrogel-coated coils caused more tissue reaction and organization compared with bare platinum coils in a rat aneurysm model, possibly owing to observed elastic lamina damage and vascular smooth muscle cell proliferation [16]. So, it may contribute to preventing recanalization in PAVM.

The so-called “3-mm guideline” is a well-known indication that recommends embolization for feeding vessels ≥ 3 mm to treat PAVM. However, there have been reports of symptomatic paradoxical embolization in patients with only sub-3-mm feeding arteries [9, 17, 18], and the recent developments of microcatheters, guidewires, and coils make it possible to treat PAVM with feeders smaller than 3 mm in diameter. As a result, the potential need to treat PAVMs in the sub-3-mm feeder range was also acknowledged by the originators of the 3-mm guideline in 2006 [19]. The 2009 hereditary hemorrhagic telangiectasia treatment guidelines now acknowledge that it is appropriate to treat PAVM with feeders smaller than 3 mm [20]. Trerotola et al. [9] has now also stated that any accessible PAVM should be treated. Thus, we have included small PAVMs that were indicated for embolization. However, in one small PAVM of our study, hydrogel-coated coils could not be placed, and conventional coils had to be used. The diameter of the feeding artery of the PAVM was 2 mm, but the minimum size of hydrogel-coated coils in our country at that time was 3 mm, and thus it was difficult to place it. However, because 2-mm hydrogel-coated coils are now available, we believe that small PAVMs can also be successfully embolized with hydrogel-coated coils. On the other hand, hydrogel-coated coils have high coil rigidity, and it can cause microcatheter kickback [21]. Thus, we think that deep advancement of a 4-Fr catheter is important for good support of the microcatheter.

In this study, we placed some coils inside the venous sac to make a scaffold so as to embolize the feeding artery tightly. It has been reported that embolization of both the feeding artery and venous sac may be useful [22–24], and some authors recommended tightly packing the venous sac [23]. However, doing it needs many coils and has a high cost. Thus, we think tight embolization of the distal feeding artery is appropriate, and placing some coils in the venous sac to make a scaffold is useful to accomplish this. In this study, the median percentage of used hydrogel-coated coils was 35% in length, but the hydrogel-coated coils could be expanded, so the median percentage of hydrogel-coated coils was 75% in volume. In addition, we aimed to place the hydrogel-coated coils at the distal feeding artery instead of the venous sac. Therefore, in the distal feeding artery, we believe the volume of hydrogel-coated coils is much higher, and thus tight embolization can be achieved efficiently. Although hydrogel-coated coils are expensive (approximately 1200 US dollars per coil), a median of 4 hydrogel-coated coils were used in our study. We think the cost is reasonable, considering the cost of re-embolization and burden that is placed on patients.

The AMPLATZER Vascular Plugs (St. Jude Medical, St. Paul, MN) is a malleable nitinol basket that forms to the shape of the vessel, and it occludes the vessel by inducing thrombus. It has been reported as a useful material for PAVM with low persistence rates (0–7%) with computed tomography (CT) evaluation [25–29]. In our country, however, AMPLATZER Vascular Plug has only recently been covered by health insurance. Therefore, further comparative studies should be necessary. The MVP Micro Vascular Plug (MVP; Covidien, Irvine, CA) is a detachable nitinol skeleton plug partially coated with polytetrafluoroethylene. Potential advantages of the MVP include microcatheter deployment, resheathability, immediate occlusion despite procedural anticoagulation, and diminished metal artifacts compared with coils on follow-up CT imaging. Initial experiences of MVP for PAVM were reported with good results [30, 31]. Unfortunately, its use has not yet been allowed in our country, but it should be a promising material because it can occlude vessels without the aid of thrombosis, and further studies should be required.

CT was routinely performed during follow-up examination, and recanalization was evaluated using the size of the draining vein and venous sac. Occlusion was diagnosed based on shrinkage after embolization, and the recanalization rate was reported to be up to 19% in some studies [4, 19, 32–34]. However, it was recently reported that TR-MRA is superior to CT for diagnosing recanalization of a PAVM, due to its high sensitivity in detecting flow and very few artifacts from platinum coils [11–14]. On the other hand, using TR-MRA, the recanalization rates are reportedly much higher than the previously reported; the rates at 3, 6, 12, and 24 months were 8, 27, 36, and 49%, respectively, for 12 untreated PAVMs, and 50, 50, 92, and 100%, respectively, for 12 recanalized PAVMs [12]. In the present study, recanalization was evaluated with TR-MRA and/or PAG, and there was no recanalization even with this more accurate evaluation method. So, we believe embolization with hydrogel-coated coils is very effective to prevent recanalization. Initial experience with hydrogel-coated coils for embolization of 9 PAVMs has been reported, with good results; the median shrinkage rate of the venous sac size was 95% on CT during a median follow-up period of 10 months (range 6–18 months) [21]. Our study would emphasize the usefulness of hydrogel-coated coils with a larger patient number, longer follow-up periods, and more reliable evaluation using TR-MRA and/or PAG.

Our study has several limitations. First, the single-centered retrospective design was a key limitation. Second, the TR-MRA equipment and spatial and temporal resolutions varied among the patients. Third, 41 of the 56 PAVMs were evaluated with TR-MRA only and were not confirmed by PAG. Forth, this is not a comparative study, so it is uncertain whether the hydrogel-coated coils are really superior to the other coils.

In conclusion, hydrogel-coated coils appear to be useful for preventing recanalization after the embolization of PAVMs; however, further prospective comparative studies are needed to investigate the long-term outcomes.
