# Correction to: Usefulness of Hydrogel-Coated Coils in Embolization of Pulmonary Arteriovenous Malformations

**DOI:** 10.1007/s00270-018-1928-x

**Published:** 2018-03-14

**Authors:** Masashi Shimohira, Tatsuya Kawai, Takuya Hashizume, Masahiro Muto, Masanori Kitase, Yuta Shibamoto

**Affiliations:** 1 Department of Radiology, Nagoya City University Graduate School of Medical Sciences, Nagoya, 467-8601 Japan; 2 Department of Radiology, Nagoya City East Medical Center, Nagoya, 464-0071 Japan; 3 Department of Radiology, Kariya Toyota General Hospital, Kariya, 448-8505 Japan

## Correction to: Cardiovasc Intervent Radiol 10.1007/s00270-018-1876-5

The article “Usefulness of Hydrogel-Coated Coils in Embolization of Pulmonary Arteriovenous Malformations,” written by Masashi Shimohira, Tatsuya Kawai, Takuya Hashizume, Masahiro Muto, Masanori Kitase and Yuta Shibamoto, was originally published electronically on the publisher’s Internet portal (currently SpringerLink) on January 17, 2018, without open access.

With the author(s)’ decision to opt for Open Choice the copyright of the article changed on March 16, 2018, to © The Author(s) 2018 and the article is forthwith distributed under the terms of the Creative Commons Attribution 4.0 International License (http://creativecommons.org/licenses/by/4.0/), which permits use, duplication, adaptation, distribution and reproduction in any medium or format, as long as you give appropriate credit to the original author(s) and the source, provide a link to the Creative Commons License and indicate if changes were made.

The original article was corrected.

